# 5-Acetyl-4-(4-methoxy­phen­yl)-6-methyl-3,4-dihydro­pyrimidin-2(1*H*)-one

**DOI:** 10.1107/S1600536808040270

**Published:** 2008-12-06

**Authors:** S. Chitra, K. Pandiarajan, N. Anuradha, A. Thiruvalluvar

**Affiliations:** a Department of Chemistry, Annamalai University, Annamalai Nagar 608 002, Tamilnadu, India; b PG Research Department of Physics, Rajah Serfoji Government College (Autonomous), Thanjavur 613 005, Tamil Nadu, India

## Abstract

In the title mol­ecule, C_14_H_16_N_2_O_3_, the heterocyclic ring adopts a flattened boat conformation, and the plane through its four coplanar atoms makes a dihedral angle of 89.65 (7)° with the benzene ring. The non-H atoms of the carbonyl, acetyl and methyl groups are nearly coplanar with the attached heterocyclic ring. Inter­molecular N—H⋯O and C—H⋯O hydrogen bonds are present in the crystal structure.

## Related literature

For chemical and medicinal background, see: Atwal *et al.* (1989 ▶); Ghorab *et al.* (2000 ▶); Kappe (1993 ▶, 2000 ▶); Kappe *et al.* (1997 ▶, 2000 ▶); Shivarama Holla *et al.* (2004 ▶); Stefani *et al.* (2006 ▶).
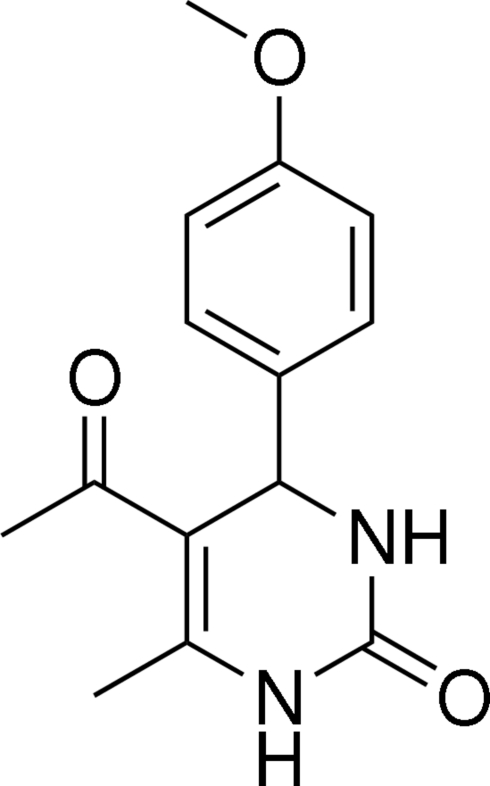


## Experimental

### 

#### Crystal data

C_14_H_16_N_2_O_3_
*M* *_r_* = 260.29
Monoclinic, 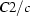

*a* = 23.7948 (12) Å
*b* = 7.9905 (3) Å
*c* = 14.4757 (7) Å
β = 108.305 (5)°
*V* = 2613.0 (2) Å^3^
*Z* = 8
Mo *K*α radiation
μ = 0.09 mm^−1^
*T* = 293 (2) K
0.3 × 0.2 × 0.2 mm

#### Data collection

Bruker Kappa APEXII CCD diffractometer
Absorption correction: multi-scan (*SADABS*; Bruker, 2004 ▶) *T*_min_ = 0.837, *T*_max_ = 1.000 (expected range = 0.821–0.981)
26518 measured reflections
2960 independent reflections
2226 reflections with *I* > 2σ(*I*)
*R*_int_ = 0.045

#### Refinement

*R*[*F* ^2^ > 2σ(*F* ^2^)] = 0.046
*wR*(*F* ^2^) = 0.149
*S* = 1.10
2960 reflections
183 parameters
H atoms treated by a mixture of independent and constrained refinement
Δρ_max_ = 0.27 e Å^−3^
Δρ_min_ = −0.24 e Å^−3^

### 

Data collection: *APEX2* (Bruker, 2004 ▶); cell refinement: *SAINT-NT* (Bruker, 2004 ▶); data reduction: *SAINT-NT*; program(s) used to solve structure: *SHELXS97* (Sheldrick, 2008 ▶); program(s) used to refine structure: *SHELXL97* (Sheldrick, 2008 ▶); molecular graphics: *ORTEP-3* (Farrugia, 1997 ▶); software used to prepare material for publication: *PLATON* (Spek, 2003 ▶).

## Supplementary Material

Crystal structure: contains datablocks global, I. DOI: 10.1107/S1600536808040270/hb2869sup1.cif

Structure factors: contains datablocks I. DOI: 10.1107/S1600536808040270/hb2869Isup2.hkl

Additional supplementary materials:  crystallographic information; 3D view; checkCIF report

## Figures and Tables

**Table 1:** Hydrogen-bond geometry (Å, °)

*D*—H⋯*A*	*D*—H	H⋯*A*	*D*⋯*A*	*D*—H⋯*A*
N1—H1⋯O15^i^	0.91 (2)	2.01 (2)	2.9209 (18)	172 (2)
N3—H3⋯O2^ii^	0.89 (2)	2.04 (2)	2.917 (2)	170.3 (19)
C16—H16*B*⋯O2^iii^	0.96	2.49	3.425 (3)	165
C61—H61*B*⋯O15^i^	0.96	2.51	3.352 (2)	146

Symmetry codes: (i) 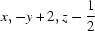; (ii) 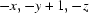; (iii) 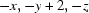.

**Fig. 1.**
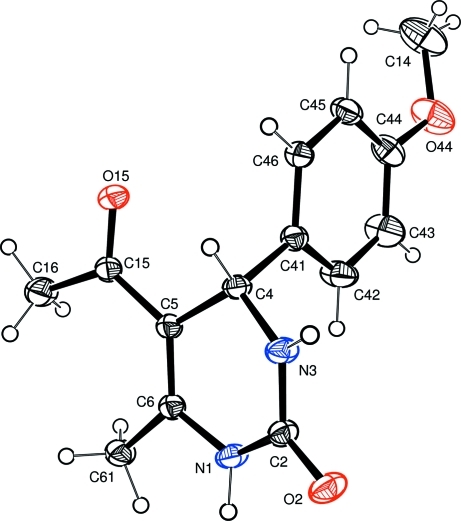
The molecular structure of (I) with displacement ellipsoids drawn at the 30% probability level. H atoms are shown as small spheres of arbitrary radius.

**Fig. 2.**
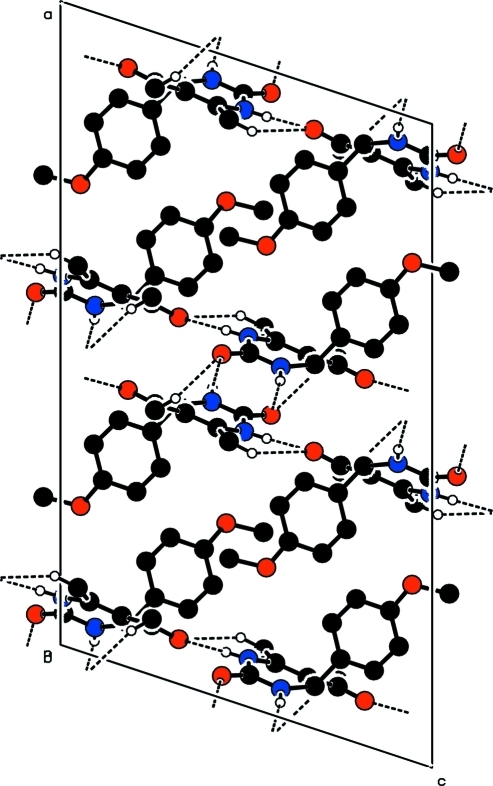
The packing of (I), viewed down the b axis. Dashed lines indicate hydrogen bonds. H atoms not involved in hydrogen bonding have been omitted.

## supplementary crystallographic information

### Comment

Dihydropyrimidinone derivatives exhibit a wide range of biological effects including antifungal, antiviral, anticancer, antibacterial, anti-inflammatory and antihypertensive effects (Kappe, 2000; Ghorab *et al.*, 2000; Shivarama Holla *et al.*, 2004). It also exhibit a biological activity of antitumour property (Kappe, 1993). Dihydropyrimidinones used as an anticancer drug capable of inhibiting Kinesin motor protein (Kappe *et al.*, 2000). Many dihydropyrimidinones and their derivatives are pharmacologically important as calcium channel blockers, antihypertensive agents and α-1a-antagonists (Atwal *et al.*, 1989; Kappe *et al.*, 1997). Dihydropyrimidin-2(1*H*)-ones can be used as an antioxidant agents (Stefani *et al.*, 2006).

In the title molecule, (I) (Fig. 1), the heterocyclic ring adopts a flattened boat conformation, and the plane through the four coplanar atoms(C2, N3, C5 and C6) makes a dihedral angle of 89.65 (7)° with the benzene ring. The carbonyl, acetyl and methyl groups, except for the H atoms, are nearly coplanar with the attached heterocyclic ring. A network of hydrogen bonds (Table 1) help to establish the packing (Fig. 2, Table 1).

### Experimental

A solution of acetylacetone (1.00 g, 0.01 mol), anisaldehyde (1.36 g, 0.01 mol) and urea (0.90 g, 0.015 mol) in EtOH (20 ml) was heated under reflux in the presence of calcium chloride (0.11 g, 0.001 mol) for 3 h (monitored by TLC). After completion of the reaction, the reaction mixture was cooled to room temperature and the reaction mixture was poured into crushed ice and the resulting solid was filtered under suction and purified by column chromatography on silica gel. Elution of 1:1 (benzene:ethyl acetate *v*/*v*) gave the product in the pure form. Yield 0.86 g (96%).

### Refinement

Atoms H1 and H3 were located in a difference map and refined isotropically. The C-bound H atoms were positioned geometrically (C—H = 0.93–0.98 Å) and refined as riding with *U*_iso_(H)= 1.2*U*_eq_(C) or 1.5*U*_eq_(methyl C).

### Crystal data

**Table:**

C_14_H_16_N_2_O_3_	*F*(000) = 1104
*M**_r_* = 260.29	*D*_x_ = 1.323 Mg m^−^^3^
Monoclinic, *C*2/*c*	Melting point: 474.5 K
Hall symbol: -C 2yc	Mo *K*α radiation, λ = 0.71073 Å
*a* = 23.7948 (12) Å	Cell parameters from 5096 reflections
*b* = 7.9905 (3) Å	θ = 2.7–26.4°
*c* = 14.4757 (7) Å	µ = 0.09 mm^−^^1^
β = 108.305 (5)°	*T* = 293 K
*V* = 2613.0 (2) Å^3^	Block, colourless
*Z* = 8	0.3 × 0.2 × 0.2 mm

### Data collection

**Table:**

Bruker Kappa APEXII CCD diffractometer	2960 independent reflections
Radiation source: fine-focus sealed tube	2226 reflections with *I* > 2σ(*I*)
graphite	*R*_int_ = 0.045
ω and φ scans	θ_max_ = 27.5°, θ_min_ = 1.8°
Absorption correction: multi-scan (*SADABS*; Bruker, 2004)	*h* = −30→30
*T*_min_ = 0.837, *T*_max_ = 1.000	*k* = −10→10
26518 measured reflections	*l* = −18→18

### Refinement

**Table:**

Refinement on *F*^2^	Primary atom site location: structure-invariant direct methods
Least-squares matrix: full	Secondary atom site location: difference Fourier map
*R*[*F*^2^ > 2σ(*F*^2^)] = 0.046	Hydrogen site location: inferred from neighbouring sites
*wR*(*F*^2^) = 0.149	H atoms treated by a mixture of independent and constrained refinement
*S* = 1.10	*w* = 1/[σ^2^(*F*_o_^2^) + (0.0771*P*)^2^ + 1.0144*P*] where *P* = (*F*_o_^2^ + 2*F*_c_^2^)/3
2960 reflections	(Δ/σ)_max_ = 0.001
183 parameters	Δρ_max_ = 0.27 e Å^−^^3^
0 restraints	Δρ_min_ = −0.24 e Å^−^^3^

### Special details

**Table:**

Geometry. Bond distances, angles *etc*. have been calculated using the rounded fractional coordinates. All su's are estimated from the variances of the (full) variance-covariance matrix. The cell e.s.d.'s are taken into account in the estimation of distances, angles and torsion angles
Refinement. Refinement of *F*^2^ against ALL reflections. The weighted *R*-factor *wR* and goodness of fit *S* are based on *F*^2^, conventional *R*-factors *R* are based on *F*, with *F* set to zero for negative *F*^2^. The threshold expression of *F*^2^ > 2σ(*F*^2^) is used only for calculating *R*-factors(gt) *etc*. and is not relevant to the choice of reflections for refinement. *R*-factors based on *F*^2^ are statistically about twice as large as those based on *F*, and *R*- factors based on ALL data will be even larger.

### Fractional atomic coordinates and isotropic or equivalent isotropic displacement parameters (Å^2^)

**Table:**

	*x*	*y*	*z*	*U*_iso_*/*U*_eq_	
O2	0.03463 (6)	0.62339 (16)	−0.06612 (9)	0.0448 (4)	
O15	0.06948 (6)	0.98745 (15)	0.31847 (8)	0.0430 (4)	
O44	0.27417 (7)	0.4726 (2)	0.44596 (12)	0.0748 (6)	
N1	0.07453 (7)	0.86574 (18)	0.00570 (10)	0.0358 (4)	
N3	0.04449 (7)	0.66399 (18)	0.09271 (10)	0.0351 (4)	
C2	0.05037 (7)	0.7104 (2)	0.00812 (11)	0.0319 (5)	
C4	0.06651 (7)	0.75657 (19)	0.18405 (11)	0.0297 (4)	
C5	0.07616 (7)	0.93953 (19)	0.16471 (11)	0.0294 (5)	
C6	0.08211 (7)	0.98438 (19)	0.07810 (11)	0.0303 (5)	
C14	0.27926 (12)	0.4564 (4)	0.54607 (19)	0.0879 (11)	
C15	0.07743 (7)	1.0494 (2)	0.24592 (11)	0.0332 (5)	
C16	0.08710 (11)	1.2339 (2)	0.24595 (14)	0.0558 (7)	
C41	0.12158 (7)	0.67712 (19)	0.25352 (11)	0.0320 (5)	
C42	0.17355 (9)	0.6594 (3)	0.23045 (14)	0.0514 (7)	
C43	0.22307 (10)	0.5896 (3)	0.29545 (17)	0.0624 (8)	
C44	0.22238 (9)	0.5391 (3)	0.38624 (14)	0.0493 (6)	
C45	0.17137 (9)	0.5551 (2)	0.41034 (13)	0.0436 (6)	
C46	0.12122 (8)	0.6231 (2)	0.34341 (12)	0.0362 (5)	
C61	0.09756 (9)	1.1526 (2)	0.04681 (13)	0.0424 (6)	
H1	0.0736 (9)	0.901 (3)	−0.0548 (16)	0.048 (6)*	
H3	0.0231 (9)	0.572 (3)	0.0913 (14)	0.046 (6)*	
H4	0.03559	0.75256	0.21553	0.0356*	
H14A	0.27276	0.56325	0.57124	0.1316*	
H14B	0.31818	0.41685	0.58148	0.1316*	
H14C	0.25029	0.37800	0.55309	0.1316*	
H16A	0.08324	1.28140	0.30456	0.0837*	
H16B	0.05821	1.28296	0.19064	0.0837*	
H16C	0.12608	1.25601	0.24265	0.0837*	
H42	0.17493	0.69534	0.17010	0.0616*	
H43	0.25728	0.57630	0.27812	0.0749*	
H45	0.17030	0.52054	0.47112	0.0523*	
H46	0.08658	0.63227	0.35988	0.0434*	
H61A	0.06570	1.22943	0.04172	0.0636*	
H61B	0.10388	1.14266	−0.01528	0.0636*	
H61C	0.13299	1.19346	0.09397	0.0636*	

### Atomic displacement parameters (Å^2^)

**Table:**

	*U*^11^	*U*^22^	*U*^33^	*U*^12^	*U*^13^	*U*^23^
O2	0.0622 (9)	0.0433 (7)	0.0320 (7)	−0.0143 (6)	0.0191 (6)	−0.0122 (5)
O15	0.0643 (9)	0.0401 (7)	0.0261 (6)	0.0044 (6)	0.0164 (6)	−0.0015 (5)
O44	0.0478 (9)	0.0956 (13)	0.0681 (11)	0.0106 (9)	−0.0003 (8)	0.0305 (10)
N1	0.0501 (9)	0.0354 (7)	0.0249 (7)	−0.0069 (6)	0.0160 (6)	−0.0019 (6)
N3	0.0469 (9)	0.0314 (7)	0.0264 (7)	−0.0105 (6)	0.0108 (6)	−0.0029 (5)
C2	0.0358 (9)	0.0334 (8)	0.0263 (8)	−0.0038 (7)	0.0094 (7)	−0.0037 (6)
C4	0.0380 (9)	0.0294 (7)	0.0233 (7)	−0.0033 (6)	0.0119 (7)	−0.0004 (6)
C5	0.0339 (9)	0.0279 (7)	0.0251 (8)	0.0007 (6)	0.0074 (6)	0.0002 (6)
C6	0.0331 (9)	0.0297 (8)	0.0266 (8)	0.0002 (6)	0.0074 (6)	−0.0003 (6)
C14	0.0716 (18)	0.102 (2)	0.0639 (17)	−0.0019 (16)	−0.0163 (13)	0.0314 (15)
C15	0.0388 (9)	0.0335 (8)	0.0240 (8)	0.0044 (7)	0.0051 (7)	−0.0013 (6)
C16	0.0959 (18)	0.0337 (9)	0.0369 (10)	−0.0012 (10)	0.0196 (11)	−0.0053 (8)
C41	0.0396 (9)	0.0275 (7)	0.0288 (8)	−0.0036 (7)	0.0108 (7)	−0.0002 (6)
C42	0.0474 (12)	0.0697 (13)	0.0400 (10)	0.0058 (10)	0.0181 (9)	0.0166 (9)
C43	0.0406 (12)	0.0861 (17)	0.0623 (14)	0.0084 (11)	0.0188 (10)	0.0201 (12)
C44	0.0436 (11)	0.0487 (11)	0.0469 (11)	0.0012 (9)	0.0017 (9)	0.0111 (9)
C45	0.0548 (12)	0.0399 (9)	0.0329 (9)	0.0014 (9)	0.0090 (8)	0.0082 (8)
C46	0.0454 (10)	0.0330 (8)	0.0314 (9)	0.0005 (7)	0.0139 (7)	0.0022 (7)
C61	0.0613 (12)	0.0324 (8)	0.0360 (9)	−0.0030 (8)	0.0188 (9)	0.0029 (7)

### Geometric parameters (Å, °)

**Table:**

O2—C2	1.235 (2)	C42—C43	1.374 (3)
O15—C15	1.228 (2)	C43—C44	1.380 (3)
O44—C14	1.422 (3)	C44—C45	1.370 (3)
O44—C44	1.371 (3)	C45—C46	1.390 (3)
N1—C2	1.373 (2)	C4—H4	0.9800
N1—C6	1.382 (2)	C14—H14A	0.9600
N3—C2	1.328 (2)	C14—H14B	0.9600
N3—C4	1.461 (2)	C14—H14C	0.9600
N1—H1	0.91 (2)	C16—H16A	0.9600
N3—H3	0.89 (2)	C16—H16B	0.9600
C4—C41	1.518 (2)	C16—H16C	0.9600
C4—C5	1.520 (2)	C42—H42	0.9300
C5—C6	1.354 (2)	C43—H43	0.9300
C5—C15	1.460 (2)	C45—H45	0.9300
C6—C61	1.501 (2)	C46—H46	0.9300
C15—C16	1.492 (2)	C61—H61A	0.9600
C41—C46	1.374 (2)	C61—H61B	0.9600
C41—C42	1.386 (3)	C61—H61C	0.9600
			
C14—O44—C44	116.75 (19)	C41—C46—C45	121.59 (18)
C2—N1—C6	123.82 (14)	N3—C4—H4	107.00
C2—N3—C4	125.54 (14)	C5—C4—H4	107.00
C2—N1—H1	114.8 (15)	C41—C4—H4	107.00
C6—N1—H1	118.3 (15)	O44—C14—H14A	109.00
C2—N3—H3	115.7 (13)	O44—C14—H14B	109.00
C4—N3—H3	118.6 (13)	O44—C14—H14C	109.00
O2—C2—N3	123.64 (16)	H14A—C14—H14B	109.00
N1—C2—N3	116.24 (14)	H14A—C14—H14C	109.00
O2—C2—N1	120.11 (15)	H14B—C14—H14C	109.00
N3—C4—C5	110.63 (13)	C15—C16—H16A	109.00
N3—C4—C41	112.17 (13)	C15—C16—H16B	109.00
C5—C4—C41	112.06 (13)	C15—C16—H16C	109.00
C4—C5—C15	113.35 (13)	H16A—C16—H16B	109.00
C6—C5—C15	127.23 (14)	H16A—C16—H16C	109.00
C4—C5—C6	119.42 (14)	H16B—C16—H16C	109.00
N1—C6—C5	119.61 (14)	C41—C42—H42	119.00
N1—C6—C61	111.81 (14)	C43—C42—H42	120.00
C5—C6—C61	128.58 (14)	C42—C43—H43	120.00
C5—C15—C16	123.96 (15)	C44—C43—H43	120.00
O15—C15—C5	118.45 (14)	C44—C45—H45	120.00
O15—C15—C16	117.59 (15)	C46—C45—H45	120.00
C4—C41—C46	119.88 (16)	C41—C46—H46	119.00
C42—C41—C46	117.89 (16)	C45—C46—H46	119.00
C4—C41—C42	122.22 (15)	C6—C61—H61A	109.00
C41—C42—C43	120.97 (19)	C6—C61—H61B	109.00
C42—C43—C44	120.4 (2)	C6—C61—H61C	109.00
O44—C44—C43	115.9 (2)	H61A—C61—H61B	109.00
C43—C44—C45	119.5 (2)	H61A—C61—H61C	109.00
O44—C44—C45	124.64 (18)	H61B—C61—H61C	109.00
C44—C45—C46	119.62 (17)		
			
C14—O44—C44—C43	165.7 (2)	C4—C5—C6—N1	5.3 (3)
C14—O44—C44—C45	−15.5 (3)	C4—C5—C6—C61	−173.60 (17)
C6—N1—C2—O2	166.26 (17)	C15—C5—C6—N1	−174.25 (17)
C6—N1—C2—N3	−12.6 (3)	C15—C5—C6—C61	6.9 (3)
C2—N1—C6—C5	12.8 (3)	C4—C5—C15—O15	−1.7 (2)
C2—N1—C6—C61	−168.20 (17)	C4—C5—C15—C16	179.10 (18)
C4—N3—C2—O2	174.99 (17)	C6—C5—C15—O15	177.84 (18)
C4—N3—C2—N1	−6.2 (3)	C6—C5—C15—C16	−1.4 (3)
C2—N3—C4—C5	21.4 (2)	C4—C41—C42—C43	178.86 (19)
C2—N3—C4—C41	−104.54 (19)	C46—C41—C42—C43	0.2 (3)
N3—C4—C5—C6	−20.2 (2)	C4—C41—C46—C45	−177.71 (15)
N3—C4—C5—C15	159.41 (15)	C42—C41—C46—C45	1.0 (3)
C41—C4—C5—C6	105.84 (17)	C41—C42—C43—C44	−1.5 (4)
C41—C4—C5—C15	−74.59 (18)	C42—C43—C44—O44	−179.4 (2)
N3—C4—C41—C42	61.2 (2)	C42—C43—C44—C45	1.7 (3)
N3—C4—C41—C46	−120.13 (16)	O44—C44—C45—C46	−179.37 (19)
C5—C4—C41—C42	−64.0 (2)	C43—C44—C45—C46	−0.5 (3)
C5—C4—C41—C46	114.72 (17)	C44—C45—C46—C41	−0.9 (3)

### Hydrogen-bond geometry (Å, °)

**Table:**

*D*—H···*A*	*D*—H	H···*A*	*D*···*A*	*D*—H···*A*
N1—H1···O15^i^	0.91 (2)	2.01 (2)	2.9209 (18)	172 (2)
N3—H3···O2^ii^	0.89 (2)	2.04 (2)	2.917 (2)	170.3 (19)
C16—H16B···O2^iii^	0.96	2.49	3.425 (3)	165
C61—H61B···O15^i^	0.96	2.51	3.352 (2)	146

Symmetry codes: (i) *x*, −*y*+2, *z*−1/2; (ii) −*x*, −*y*+1, −*z*; (iii) −*x*, −*y*+2, −*z*.
